# Supplementary material for: Non-infectious diseases in infectious disease consultation: A descriptive study in a tertiary care teaching hospital
Source: PLoS One. 2023 Dec 8;18(12):e0295708. doi: 10.1371/journal.pone.0295708 (PMC10707697; doi:10.1371/journal.pone.0295708)
Supplement: Attachment — Submitted filename: 231013_response_to_reviewer.docx [file pone.0295708.s002.docx]

PONE-D-23-27435

Non-infectious diseases in infectious disease consultation: A descriptive study in a tertiary care teaching hospital

PLOS ONE

Dear Dr. Hadano,

Thank you for submitting your manuscript to PLOS ONE. After careful consideration, we feel that it has merit but does not fully meet PLOS ONE’s publication criteria as it currently stands. Therefore, we invite you to submit a revised version of the manuscript that addresses the points raised during the review process.

Please submit your revised manuscript by Nov 17 2023 11:59PM. If you will need more time than this to complete your revisions, please reply to this message or contact the journal office at plosone@plos.org. When you're ready to submit your revision, log on to https://www.editorialmanager.com/pone/ and select the 'Submissions Needing Revision' folder to locate your manuscript file.

Please include the following items when submitting your revised manuscript:

A rebuttal letter that responds to each point raised by the academic editor and reviewer(s). You should upload this letter as a separate file labeled 'Response to Reviewers'.

A marked-up copy of your manuscript that highlights changes made to the original version. You should upload this as a separate file labeled 'Revised Manuscript with Track Changes'.

An unmarked version of your revised paper without tracked changes. You should upload this as a separate file labeled 'Manuscript'.

If you would like to make changes to your financial disclosure, please include your updated statement in your cover letter. Guidelines for resubmitting your figure files are available below the reviewer comments at the end of this letter.

If applicable, we recommend that you deposit your laboratory protocols in protocols.io to enhance the reproducibility of your results. Protocols.io assigns your protocol its own identifier (DOI) so that it can be cited independently in the future. For instructions see: https://journals.plos.org/plosone/s/submission-guidelines#loc-laboratory-protocols. Additionally, PLOS ONE offers an option for publishing peer-reviewed Lab Protocol articles, which describe protocols hosted on protocols.io. Read more information on sharing protocols at https://plos.org/protocols?utm_medium=editorial-email&utm_source=authorletters&utm_campaign=protocols.

We look forward to receiving your revised manuscript.

Kind regards,

Tai-Heng Chen, M.D.

Academic Editor

PLOS ONE

Journal requirements:

1. When submitting your revision, we need you to address these additional requirements.

Please ensure that your manuscript meets PLOS ONE's style requirements, including those for file naming. The PLOS ONE style templates can be found at

https://journals.plos.org/plosone/s/file?id=wjVg/PLOSOne_formatting_sample_main_body.pdf and

https://journals.plos.org/plosone/s/file?id=ba62/PLOSOne_formatting_sample_title_authors_affiliations.pdf

2. We noticed you have some minor occurrence of overlapping text with the following previous publication(s), which needs to be addressed:

https://journals.lww.com/md-journal/Fulltext/2020/06190/Infectious_disease_consultations_and_newly.84.aspx

https://www.nature.com/articles/s41598-022-23392-6

3. In your revision ensure you cite all your sources (including your own works), and quote or rephrase any duplicated text outside the methods section. Further consideration is dependent on these concerns being addressed.

Thank you for your feedback. We have reviewed the mentioned previous publications, and we acknowledged the minor occurrence of overlapping text. We have addressed this issue in the revised manuscript. In the revision, we have ensured that all sources, including our own work, are appropriately cited, and any duplicated text outside the methods section have been either quoted or rephrased as necessary. We appreciate your feedback and will ensure that these concerns are fully addressed in the revised version of the manuscript.

[Note: HTML markup is below. Please do not edit.]

Reviewers' comments:

Reviewer's Responses to Questions

Comments to the Author

1. Is the manuscript technically sound, and do the data support the conclusions?

The manuscript must describe a technically sound piece of scientific research with data that supports the conclusions. Experiments must have been conducted rigorously, with appropriate controls, replication, and sample sizes. The conclusions must be drawn appropriately based on the data presented.

Reviewer #1: Yes

Reviewer #2: Yes

2. Has the statistical analysis been performed appropriately and rigorously?

Reviewer #1: Yes

Reviewer #2: Yes

3. Have the authors made all data underlying the findings in their manuscript fully available?

The PLOS Data policy requires authors to make all data underlying the findings described in their manuscript fully available without restriction, with rare exception (please refer to the Data Availability Statement in the manuscript PDF file). The data should be provided as part of the manuscript or its supporting information, or deposited to a public repository. For example, in addition to summary statistics, the data points behind means, medians and variance measures should be available. If there are restrictions on publicly sharing data—e.g. participant privacy or use of data from a third party—those must be specified.

Reviewer #1: No

Reviewer #2: No

Thank you for your comment. We have added the dataset in the revised manuscript.

4. Is the manuscript presented in an intelligible fashion and written in standard English?

PLOS ONE does not copyedit accepted manuscripts, so the language in submitted articles must be clear, correct, and unambiguous. Any typographical or grammatical errors should be corrected at revision, so please note any specific errors here.

Reviewer #1: Yes

Reviewer #2: Yes

5. Review Comments to the Author

Please use the space provided to explain your answers to the questions above. You may also include additional comments for the author, including concerns about dual publication, research ethics, or publication ethics. (Please upload your review as an attachment if it exceeds 20,000 characters)

We appreciate your valuable feedback, which will help us refine our manuscript and contribute to the overall quality of our research. Thank you for your time and consideration.

Reviewer #1: Thank you for the opportunity to review this insightful paper.

This study investigates the non-infectious disease component of infectious disease consultations in acute care hospitals in Japan.

From the standpoint of enhancing the quality of ID consultations, this research is invaluable.

However, before publication, certain revisions are necessary.

Major comments:

1)The authors mention that the multiple logistic regression analysis revealed that "Diagnosis and management," "Internal medicine consultation," and "non-bacteremia cases" were independently associated with non-ID cases.

Yet, there is no subsequent discussion on these three outcomes.

It is imperative for the authors to address them.

Thank you for your insightful comments. We have provided additional comment to the Discussion section (Discussion section, lines 187-196, page 14). This change has been made.

2)While the authors elaborate on the distribution of non-ID cases over the period, there is a lack of detailed information regarding the ID cases.

If feasible, a comprehensive description of the ID cases would be beneficial.

Thank you for your insightful comment. I have added the top 5 infectious diseases diagnosed at consultation to the Results section (lines 118-121, page 8).This change has been made.

Minor comments:

Table 3:

The figures in the category of connective tissue and collagen vascular diseases are inconsistent.

What does the ")" signify in "oesteitis)?" The ")" appears to be an careless mistake by the author.

Regarding the inconsistency in the figures for the category of connective tissue and collagen vascular diseases, we have thoroughly reviewed our data and made necessary corrections to ensure accuracy in the table.

Regarding the ")" in "oesteitis)," it appears to be a typographical error. We have corrected this appropriately to read "osteitis" .

This change has been made.

Reviewer #2: The manuscript is a single-centre retrospective review of noninfectious diseases in patients who received an infectious diseases (ID) consult. The manuscript is clearly written, the conclusions are sound, and the main limitations are correctly summarised.

The article's main limitation is a lack of generalisability. The centre where patients were recruited lacks a general medical department. Thus the pattern of non-infectious diagnoses for which ID were consulted may be very different to that in a hospital where generalists (internists, etc.) are more commonplace, and who may be more comfortable making non-infectious diagnoses. The authors do acknowledge this in the limitations section, but it remains a significant limitation nonetheless.

We appreciate your thorough review of our manuscript and your acknowledgment of its clarity and sound conclusions. We also value your insight regarding the main limitation, which is the lack of generalizability.

To address this concern, we have added the following statement to the limitations section: "It is necessary to consider the possibility of conducting a multi-center study to improve the generalizability of the research."

I have only minor comments:

1. I wasn't clear in the methods section how patients with both an ID and a non-ID diagnosis were categorised. (E.g. a patient with Hodgkins Lymphoma and also a bacteraemia). Greater clarity on this point would be welcome, since it speaks to whether the ID consultation was appropriate or not.

Thank you for your valuable feedback. We initially categorized patients as either ID or non-ID based on the final diagnosis in the database. However, it has come to our attention that some cases involved a combination of both, and there were discrepancies in some of the data.

To address this issue, we have now divided patients into three groups: ID, non-ID, and those with a combination of both. We have also added a definition in the Methods section. Consequently, there have been modifications to both the aggregated results and the analysis results. Additionally, in the section related to tumors, we have provided a breakdown distinguishing between 1) Neoplastic fever caused by known tumors and 2) the diagnosis of new tumors.

2. In the abstract, lines 33-37 are largely redundant, since they restate the preceding sentences.

Thank you for your feedback. Lines 33-37 have been removed. This change has been made.

3. Findings such as ID consultation for "diagnosis and management [as opposed to for treatment of established infections] was more common in the non-ID group" are almost inevitable from the definitions used (if the ID problem was obvious, then there would be no reason to consult the ID division for diagnosis). This may be worth clarifying in the discussion.

Thank you for your feedback. I agree with the difficulty with the case definition. I have added an update to the discussion section.

6. PLOS authors have the option to publish the peer review history of their article (what does this mean?). If published, this will include your full peer review and any attached files.

If you choose “no”, your identity will remain anonymous but your review may still be made public.

Do you want your identity to be public for this peer review? For information about this choice, including consent withdrawal, please see our Privacy Policy.

Reviewer #1: No

Reviewer #2: No
